# Geographic Variability and Anti-Staphylococcal Activity of the Chrysophaentins and Their Synthetic Fragments

**DOI:** 10.3390/md10051103

**Published:** 2012-05-22

**Authors:** Jessica L. Keffer, Jared T. Hammill, John R. Lloyd, Alberto Plaza, Peter Wipf, Carole A. Bewley

**Affiliations:** 1 Laboratory of Bioorganic Chemistry, National Institute of Diabetes and Digestive and Kidney Diseases, National Institutes of Health, Bethesda, MD 20892, USA; Email: kefferj@mail.nih.gov (J.L.K.); lloydj@niddk.nih.gov (J.R.L.); a.plaza@mx.uni-saarland.de (A.P.); 2 Center for Chemical Methodologies and Library Development, University of Pittsburgh, Pittsburgh, PA 15260, USA; Email: jth25@pitt.edu (J.T.H.); pwipf@pitt.edu (P.W.)

**Keywords:** chrysophaentins, *Staphylococcus aureus*, antibacterial

## Abstract

Drug-resistant *Staphylococcus aureus* is a continuing public health concern, both in the hospital and community settings. Antibacterial compounds that possess novel structural scaffolds and are effective against multiple *S. aureus* strains, including current drug-resistant ones, are needed. Previously, we have described the chrysophaentins, a family of bisdiarylbutene macrocycles from the chrysophyte alga *Chrysophaeum taylori* that inhibit the growth of *S. aureus* and methicillin-resistant *S. aureus* (MRSA). In this study we have analyzed the geographic variability of chrysophaentin production in *C. taylori* located at different sites on the island of St. John, U.S. Virgin Islands, and identified two new linear chrysophaentin analogs, E2 and E3. In addition, we have expanded the structure activity relationship through synthesis of fragments comprising conserved portions of the chrysophaentins, and determined the antimicrobial activity of natural chrysophaentins and their synthetic analogs against five diverse *S. aureus* strains. We find that the chrysophaentins show similar activity against all *S. aureus* strains, regardless of their drug sensitivity profiles. The synthetic chrysophaentin fragments indeed mimic the natural compounds in their spectrum of antibacterial activity, and therefore represent logical starting points for future medicinal chemistry studies of the natural products and their analogs.

## 1. Introduction

*Staphylococcus aureus* has long been a public health concern, and is currently the leading cause of skin, soft tissue, bloodstream, and lower respiratory tract bacterial infections [1]. Penicillin was discovered in 1928 and first used in 1941, but by the late 1940’s was ineffective due to the rise in penicillinase-producing *S. aureus* [2,3]. Methicillin was introduced into the clinic to combat penicillin-resistant strains, but not long after its introduction, the first case of methicillin-resistant *S. aureus* (MRSA) was described [4,5]. In the late 1980s and 1990s, the proportion of methicillin resistant infections rose significantly compared to susceptible ones worldwide [6]. In the United States, *S. aureus* is the leading cause of hospital-acquired infections [7]. Most of these strains are methicillin-resistant, and in the early 2000’s, more patients in the U.S. succumbed to lethal MRSA infections than to AIDS, tuberculosis, and hepatitis B combined [8].

The public health threat involving MRSA is not just limited to the spread of antibacterial resistance; the movement of MRSA infections out of hospital settings and into the community is of great concern. These community-associated MRSA (CA-MRSA) strains have become so prevalent in the United States that almost everyone can be considered at risk for these infections [9,10]. CA-MRSA infections present unique challenges to their treatment because they are distinct from hospital-acquired MRSA (HA-MRSA) from an epidemiological, genetic, and clinical perspective [11,12]. CA-MRSA strains generally have a faster growth rate than hospital acquired strains, but are less resistant to antibacterials [13]. These bacteria typically contain a type IV staphylococcal cassette chromosome *mec* type IV gene (SCC *mec* IV) and are capable of producing the extracellular cytotoxin Panton-Valentine leukocidin (PVL) [14,15]. In contrast, nosocomial *S. aureus* typically carry a type I, II, or III staphylococcal cassette chromosome *mec*. CA-MRSA is fully virulent, perhaps even more so than HA-MRSA, and can produce other toxins that vary by geographic location [16,17]. In the United States, there are two distinct community-associated backgrounds that comprise the majority of CA-MRSA infections, namely USA300 and USA400. USA400 was first identified, but for reasons not completely understood, USA300 has become the predominant infectious CA-MRSA clone [7,18,19], currently accounting for 90–95% of CA-MRSA outpatient isolates in the US [13,20,21]. One reason for its relative success and apparent fitness advantage may be its increased virulence. In a rat pneumonia model, researchers have shown increased pathogenicity that correlates with increased expression of virulence genes and increased expression of *S. aureus* regulatory systems [17,22].

The decline in antibacterial drug discovery programs worldwide has led to a concurrent decrease in the number of new antibacterial compounds [23]. This decline is apparent from the decrease in the number of antibacterial drugs (not including vaccines) approved for use from 1981–2010. During this period, 56 drugs were approved from 1981–1990, 32 were approved in 1991–2000, and only 16 were approved from 2001–2010 [24]. The increasing prevalence of resistance, even to drugs of last resort such as vancomycin, indicates that more new compounds are still needed [25,26]. These trends, combined with the knowledge that while incremental changes to lead compound scaffolds that result in increased potency and spectrum generally do not provide a permanent solution to combating resistance, suggest that new antibiotic compounds should differ structurally from earlier generation antibacterials. 

Previously, we reported a suite of novel bisdiarlybutene macrocycles with antibacterial activity isolated from the marine chrysophyte alga, *Chrysophaeum taylori*, from St. John, U.S. Virgin Islands [27]. We found that these compounds have inhibitory activity against three drug-resistant Gram-positive organisms, and several drug-susceptible strains, and we developed a structure-activity relationship to explain their potencies. We made a subsequent collection from three locations on St. John, and have looked at geographic and temporal variability of chrysophaentin production. Furthermore, to address supply limitations of the natural products, we are working towards a total synthesis of the chrysophaentins, and have in hand two fragments from this effort. In this study, we have expanded the antibacterial activity and structure activity relationship of the natural chrysophaentins and two synthetic fragments to two additional *S. aureus* strains, *S. aureus* UAMS-1, a clinical osteomyelitis isolate, and CA-MRSA USA300-LAC. It was important to expand our library of *S. aureus* strains to include one with a more recent clinical, but still drug-susceptible, background, and a drug-resistant strain comprising the predominant and successfully invasive CA-MRSA USA300. We investigated the natural and synthetic chrysophaentin antibacterial activity to determine overall potency of growth inhibition and the mode of inhibition (bacteriostatic or bactericidal).

## 2. Results and Discussion

### 2.1. *Chrysophaeum taylori* Collection and Chrysophaentin Identification

**Figure 1:**
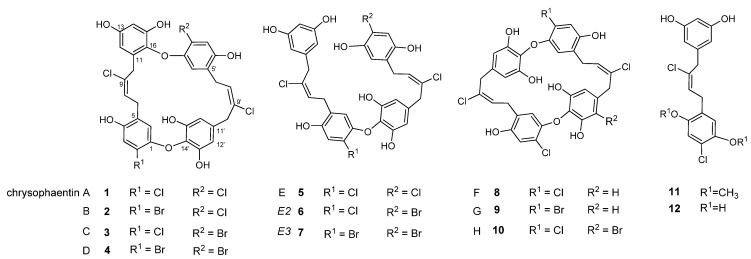
Structures of the natural chrysophaentins and synthetic fragments.

The original chrysophaentins came from one collection of *C. taylori* made in Round Bay, located on the southern side of St. John, U.S. Virgin Islands, in July 2007. From this collection, we identified four asymmetrically linked macrocycles, chrysophaentins A–D (**1–4**); one linear analog, chrysophaentin E (**5**); and three symmetrically-linked macrocycles, chrysophaentins F–H (**8–10**) (Figure 1). The eight chrysophaentins were remarkably similar to one another. All possess an ether linkage between C-1 and C-14′. If present, the second ether linkage was located between either C-14 or C-16 and C-1′, and halogen variability only occurred at C-2 and C-2′. Chrysophaentin H additionally differed in its pentahalogenation, with replacement of the hydrogen at C-12′ with a bromine.

**Figure 2:**
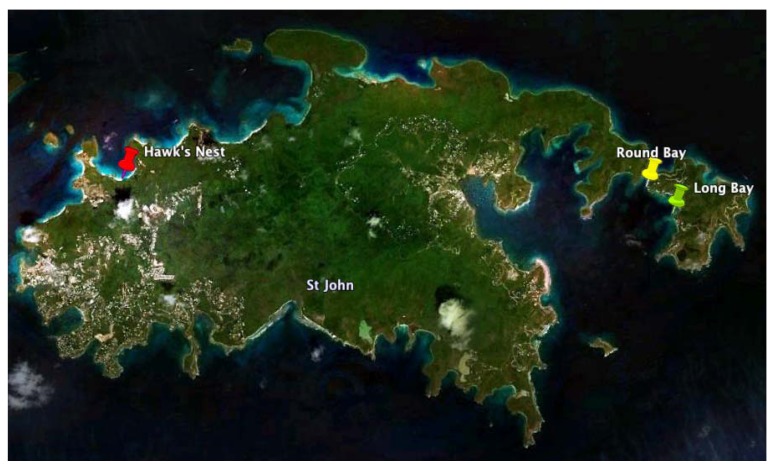
Locations of *Chrysophaeum taylori* collections on St. John, U.S. Virgin Islands. Collections were made Long Bay in 2009 (green pin), the northern tip of Round Bay in 2007 and 2009 (yellow pin), and on the north side of the island at Hawk’s Nest in 2009 (red pin). Image was created using the Google Earth software package [28].

**Figure 3:**
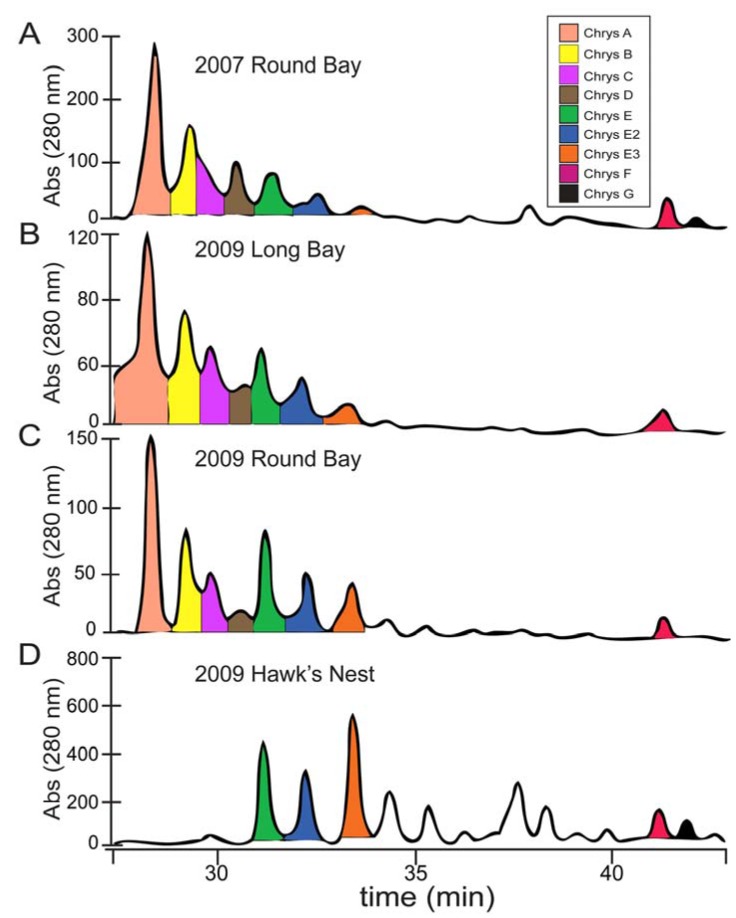
Geographic distribution of chrysophaentins determined by LCMS. Analytical HPLC chromatograms of MeOH extracts of four collections of *Chrysophaeum taylori* eluting with a linear gradient of 50–80% MeOH in aq. 0.05%TFA in 50 min. Panels correspond to *C. taylori* extracts obtained from the following collections sites and dates: (**A**) Round Bay, 2007; (**B**) Long Bay, 2009; (**C**) Round Bay, 2009; (**D**) Hawk’s Nest, 2009. The expansions shown correspond to the chromatograms at 28–42 min, and UV detection at 280 nm. Peaks are colored as shown in the legend, panel A.

To obtain additional raw material, we returned to St. John in June 2009 and made three collections at Round Bay, Long Bay, and Hawk’s Nest (Figure 2). Each sample was dried and sequentially extracted with hexanes, chloroform, and methanol, and the chrysophaentins were found to be present in the methanol extract. Each methanolic extract from the three new collections plus the original collection were chromatographed by reverse-phase HPLC on a Jupiter Proteo C12 column with a linear gradient of 50–80% methanol in 0.05% TFA-water in fifty minutes. This afforded a chromatographic picture of the chrysophaentins present in each extract (Figure 3). The two collections from Round Bay (Figure 3A,C), separated by two years, were remarkably similar, as was the collection from Long Bay (Figure 3B). However, it was immediately obvious that the methanol extract from Hawk’s Nest (Figure 3D) was different; in fact, it lacked the asymmetrically-linked chrysophaentins A–D. Instead, the linear chrysophaentin E was the most abundant, as well as two newly identified linear chrysophaentins, termed E2 and E3 (Figure 1, compounds **6** and **7**). Algae collected from all locations contained the symmetrical chrysophaentins, albeit at much lower abundance relative to compounds A–E.

### 2.2. Identification of New Acyclic Chrysophaentins

**Figure 4:**
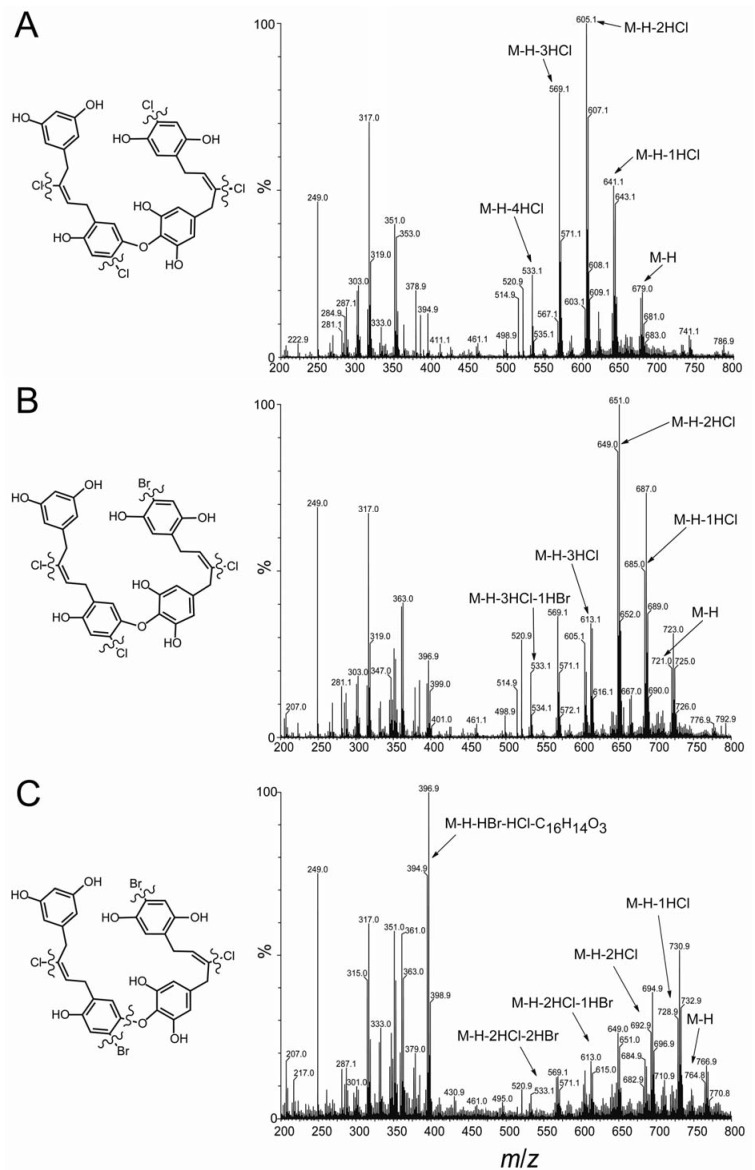
HR-MS fragmentation of chrysophaentins E, E2, and E3. Fragmentation of (**A**) chrysophaentin E showing individual losses of four chlorine atoms; (**B**) chrysophaentin E2 showing loss of three chlorine atoms and one bromine atom; (**C**) the major ion in the spectrum for chrysophaentin E3 corresponds to fragmentation at the ether bond, with weaker ions corresponding to loss of two chlorine and two bromine atoms.

Previously we determined the structure of acyclic chrysophaentin E (**5**), which has a molecular formula of C_32_H_26_Cl_4_O_8_ as determined by HR-MS and NMR. Chrysophaentins E2 (**6**) and E3 (**7**) were obtained in insufficient quantities for full NMR characterization. However, through analysis of the respective fragmentation patterns observed in the HR-ESI-MS data, we were able to assign structures for each of these new linear chrysophaentins. HR-ESI-MS showed chrysophaentin E2 to possess a molecular formula of C_32_H_26_BrCl_3_O_8_ (*m/z* 720.9786 [M − H]^−^, calcd for C_32_H_25_BrCl_3_O_8_, 720.9798), and chrysophaentin E3 to possess a molecular formula of C_32_H_26_Br_2_Cl_2_O_8_ (*m/z* 764.9282 [M − H]^−^, calcd for C_32_H_25_Br_2_Cl_2_O_8_, 764.9293), indicating that a bromine replaced a chlorine in E2 (**6**), and two bromines replaced two chlorines in E3 (**7**), when compared to chrysophaentin E (**5**). Since positions C-2 and C-2′ were the only sites of halogen variability in the natural chrysophaentins, we reasoned that the dibrominated compound **7** likely contained a bromine at both of these carbons. On the other hand, chrysophaentin E2 must contain bromine at only one of these sites, with chlorine at the other. To identify the respective locations of chlorine and bromine in **6**, we compared the mass fragmentation patterns of the three linear analogs (Figure 4). As seen in Figure 4A, the fragmentation pattern for chrysophaentin E showed the successive loss of four chlorine atoms, giving fragment ions with clear isotopic signatures at *m/z* 641 [M − H − HCl]^−^, 605 [M − H − 2HCl]^−^, 569 [M − H − 3HCl]^−^, and 533 [M − H − 4HCl]^−^. The fragmentation pattern and peak intensities for chrysophaentin E2 (**6**) was very similar to **5** (Figure 4A and Figure 4B), where successive loss of three chlorines and one bromine gave rise to fragment ions at *m/z* 685 [M − H − HCl]^−^, 649 [M − H − 2HCl]^−^, 613 [M − H − 3HCl]^−^, and 533 [M − H − HBr − 3HCl]^−^. In contrast, although loss of two bromines and two chlorines was apparent in the mass spectrum for chrysophaentin E3, the major ion for **7** appeared at *m/z* 395 [M − H − HBr − HCl − C_16_H_14_O_3_]^−^, which corresponds to the fragment generated from cleavage at the ether bond (Figure 4C). Although MS data on model halogenated biaryl ethers was not available, our data suggest that the presence of bromine *ortho* to the ether bond is destabilizing as compared to chlorine in the same position, and facilitates cleavage at the ether bond in compound **7**. Thus, the nearly identical mass spectral patterns observed for **5** and **6**, and the unique fragmentation at the ether bond in the spectrum for **7**, allow assignments of the structures of the new linear chrysophaentins E2 and E3, as shown in Figure 1 and Figure 4.

### 2.3. Chrysophaentin Fragment Synthesis

**Scheme 1:**
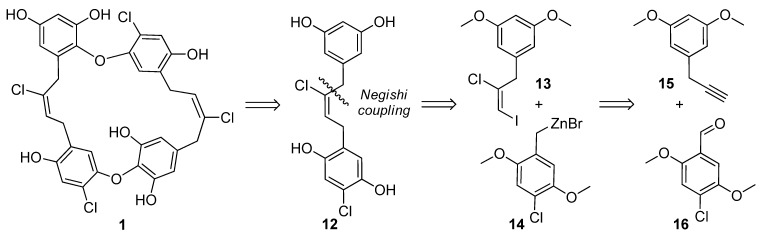
Retrosynthetic approach toward chrysophaentin A (**1**).

While the extracts from Long Bay and Round Bay afforded more of the asymmetrical chrysophaentins, it became apparent that a synthetic approach to chrysophaentin production was necessary in order to fully examine the biological activity of the structural variants. Accordingly, we devised a convergent synthetic strategy to allow for a total synthesis of the chrysophaentins as well as the investigation of structure-activity relationships. Fragment **12**, which, in principle, could be used twice for the assembly of the diether **1**, could be constructed by a key Negishi cross-coupling between vinyl iodide **13** and benzylic zinc species **14** (Scheme 1). A chloroiodination of alkyne **15** and a metalation of a halide derived from aldehyde **16** were envisioned to provide access to **13** and **14**, respectively. In fact, while some modification of protective groups was required, this strategy could be readily realized.

**Scheme 2:**
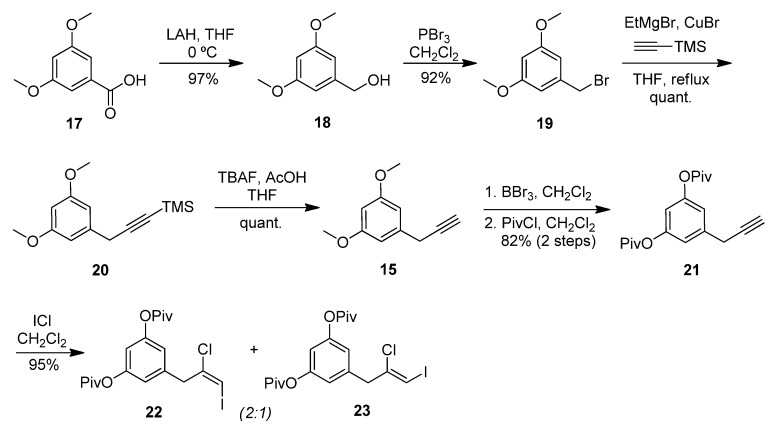
Preparation of (*E*)-iodoalkene segment (**22**).

The commercially available benzoic acid **17** was reduced with lithium aluminumhydride (LAH) to the benzylalcohol **18** (Scheme 2). Bromination [29] and copper-catalyzed substitution with TMS-acetylene magnesium bromide [30] provided the alkyne **20** in 89% overall yield from **17**. After desilylation of **20** with tetrabutylammonium fluoride (TBAF), dimethyl resorcinol **15** was subjected to iodochlorination [31,32] in an attempt to generate **13**. However, the desired alkyne addition product was formed to a minor extent, and mainly overiodination products were observed. Therefore, we demethylated **15** with boron tribromide and installed the deactivating pivaloyl esters in 82% yield. Iodochlorination of **21** now proceeded in excellent yield to give a 2:1 mixture of alkene isomers **22** and **23**. While this ratio could be further improved in favor of the (*E*)-alkene **22** using solvents other than dichloromethane, yields were considerably lower. Furthermore, we were unable to separate the mixture at this stage, and the clean conversion in dichloromethane allowed us to carry it forward to the coupling step.

**Scheme 3:**
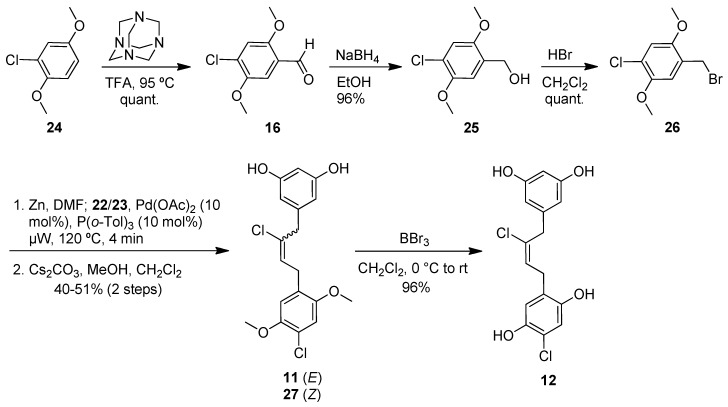
Preparation of benzyl bromide segment (**26**) and Negishi coupling to give tetraphenol **12**.

Formylation [33,34] of dimethyl hydroquinone **24** provided aldehyde **16**, which was reduced to the alcohol **25** and brominated to give **26** in high yield (Scheme 3). Four equivalents of this bromide were then converted to the corresponding zinc reagent. Removal of excess zinc was necessary prior to combination with **22** and **23** and subsequent microwave heating in the presence of palladium acetate. These cross-coupling conditions [35] generated satisfactory yields of the chloroalkenes **11** and **27** in a 2:1 ratio after methanolysis of the pivaloate esters with cesium carbonate and methanol. An analytically pure sample of **11** was obtained by separation with supercritical fluid chromatography (SFC). Boron tribromide was used to cleave the two methyl ethers on **11** to give the tetraphenol **12**, which was obtained as the pure (*E*)-isomer and further purified by preparative supercritical fluid chromatography (SFC) before submission to biological testing. In a control experiment, the use of a 2:1 mixture of **11 **and **27 **in the deprotection reaction led to a 2:1 mixture of **12 **and its (*Z*)-diastereomer, as expected. The assignments of the *E/Z*-configurations of **12** and its (*Z*)-diasteromer as well as **22** and **23** were mainly based on the available literature data [31,32].

### 2.4. Antibacterial Evaluation

With the new fragments in hand, we tested whether their potencies were similar to the parent chrysophaentins. A microbroth dilution assay was used to evaluate the antibacterial activity of these compounds, and the minimum inhibitory concentration that led to a 50% decrease in growth (MIC_50_) was determined using a model curve fit to the data. The MIC_90_ was determined as the lowest concentration where there was no visible growth, and the minimum bactericidal concentration (MBC) was determined as the lowest concentration where a plated aliquot of treated bacteria led to a three logarithmic decrease in colony forming units (CFU) per milliliter relative to the starting inoculum. All compounds were evaluated against three different *S. aureus* strains from ATCC and two diverse clinical isolates. The strains used in this study included: *S. aureus* ATCC 25923, a clinical isolate from Seattle, 1945; methicillin-resistant *S. aureus* ATCC BAA-41, a hospital-acquired strain isolated in New York City in 1994; multidrug-resistant *S. aureus* (MDRSA) ATCC BAA-44, a hospital-acquired strain isolated in Lisbon, Portugal, with resistance towards ampicillin, methicillin, oxacillin, penicillin, erythromycin, gentamicin, tetracycline, azithromycin, amikacin, clindamycin, cephalothin, ceftriaxone, imipenem, lincomycin, streptomycin, perfloxacin, rifampin, and neomycin [6,36]; *S. aureus* UAMS-1, a clinical osteomyelitis isolate [37]; and community-associated methicillin-resistant *S. aureus*, USA300-LAC [38,39].

#### 2.4.1. Minimum Bactericidal Concentrations for Select Natural Chrysophaentins

The MIC_50_ for select chrysophaentins was reported previously [27]. Those qualitative antibacterial data have been expanded here to include descriptions of the MIC_90_ and the MBC for the chrysophaentins against *S. aureus* 25923, MRSA BAA-41, and MDRSA BAA-44. These values are summarized in Table 1. We found chrysophaentin A to inhibit the growth of *S. aureus* 25923, MRSA BAA-41 and MDRSA BAA-44 with MIC_50_ values on the order of 1–4 μM, and MIC_90_ values ranging from 4–9 µM against all three strains. Chrysophaentin A was bacteriostatic at its MIC_90_, becoming bactericidal only at concentrations four to eight times higher than the MIC_90_. We have found chrysophaentin A to be the most potent compound in all of our antibacterial assays, yet chrysophaentins F and H which bear a symmetrical bisbibenzyl ether skeleton also inhibit *S. aureus* 25923 and MRSA BAA-41 with single digit micromolar MIC_50_s. While their MIC_90_s were higher than those of chrysophaentin A (17–19 µM), the MBC for chrysophaentin F was still four-fold higher than the MIC_90_. A significant reduction in potency was observed for the acyclic natural product chrysophaentin E (**5**), which was also bacteriostatic at its MIC_90_. Chrysophaentins D (**4**) and G (**9**), both of which contain a bromine atom on the D ring, were the least active compounds, with an 8- to13-fold increase in MIC_50_s values, and an 8-fold increase in MIC_90_s.

**Table 1:** Antibacterial summary of select chrysophaentins and synthetic fragments against five diverse strains of *Staphylococcus aureus* (all values are in µM; n.d. means not determined).

	*S. aureus* 25923			MRSA BAA-41			MDRSA BAA-44		*S. aureus* UAMS-1			CA-MRSA USA300-LAC		
	MIC_50_	MIC_90_	MBC	MIC_50_	MIC_90_	MBC	MIC_50_	MIC_90_	MIC_50_	MIC_90_	MBC	MIC_50_	MIC_90_	MBC
**1**	2.7 ± 0.9	9.2	37	2.3 ± 1.0	4.6	37	1.8 ± 0.5	9.2	5.1 ± 2.1	9.2	19	5.0 ± 2.4	9.2	19
**4**	37 ± 16	65	n.d.	26 ± 8.5	65	n.d.	n.d.	n.d.	49 ± 23	65	>65	41 ± 20	65	>65
**5**	16 ± 5.3	37	74	14 ± 4.1	37	74	n.d.	n.d.	n.d.	n.d.	n.d.	n.d.	n.d.	n.d.
**8**	7.9 ± 2.9	19	74	6.3 ± 1.9	19	74	n.d.	n.d.	12 ± 5.6	37	>74	12 ± 5.1	37	74
**9**	23 ± 7.5	69	n.d.	17 ± 4.3	69	n.d.	n.d.	n.d.	n.d.	n.d.	n.d.	n.d.	n.d.	n.d.
**10**	5.9 ± 1.8	17	n.d.	6.2 ± 1.9	17	n.d.	n.d.	n.d.	n.d.	n.d.	n.d.	n.d.	n.d.	n.d.
**11**	12 ± 4.3	34	68	11 ± 5.4	34	68	13 ± 4.7	34	20 ± 10	34	68	18 ± 8.4	34	68
**12**	20 ± 5.2	74	150	23 ± 9.9	74	150	27 ± 9.4	74	31 ± 13	74	150	29 ± 9.9	74	150

#### 2.4.2. Antibacterial Activity of Synthetic Chrysophaentin Fragments

We were gratified to find that compound **11** inhibited the growth of *S. aureus* 25923 with MIC_50_ and MIC_90_ values comparable to those for the linear and symmetrically linked natural products **5** and **8** (Table 1). At the MIC_90_, compound **11** was bacteriostatic, and its MBC was 68 µM. Compound **11** also inhibited the growth of MRSA BAA-41 and MDRSA BAA-44 with low micromolar MIC_50_s, and an MIC_90_ of 34 µM for both strains. Compound **12** was slightly less active with MIC_50_s in the range of 20–28 μM against *S. aureus* 25923, MRSA BAA-41, and MDRSA BAA-44. An increase to 74 µM in MIC_90_ was observed for all three strains. Compound **12** was bacteriostatic at this concentration, with a MBC of ~150 µM. 

#### 2.4.3. Antibacterial Activity against *S. aureus* UAMS-1 and CA-MRSA USA300-LAC

Given the notable antibacterial activity observed for diverse laboratory strains of *S*. *aureus*, we were interested to compare the effects of several representative compounds on the clinical *S. aureus* strains UAMS-1 and CA-MRSA USA300-LAC. Compounds evaluated included chrysophaentins A, D, and F, and synthetic compounds **11** and **12**, and MIC_50_, MIC_90_ and MBC values were determined for each compound. Chrysophaentin A was again the most potent, with an MIC_50_ of 5 µM for both clinical strains (Table 1), and the MIC_90_ was only slightly higher at 9 µM. Chrysophaentin A was still bacteriostatic at low micromolar concentrations, and the MBC was only 2 times higher at 19 µM. By comparison, the symmetrical chrysophaentin F (**8**) was slightly less active with respective MIC_50_ and MIC_90_ values of 12 and 37 µM, and was also bacteriostatic at the MIC_90_. The MIC_50_s for compounds **11** and **12** were similar to those of **8** as was the MIC_90_ for compound **11**, while the MIC_90_ for compound **12** was higher. For both synthetic compounds, the MBC was two-fold higher than the MIC_90_. Finally, chrysophaentin D (**4**) was the least active with MIC_50_s between 41 and 49 µM, and MIC_90_s at 65 µM. 

**Figure 5:**
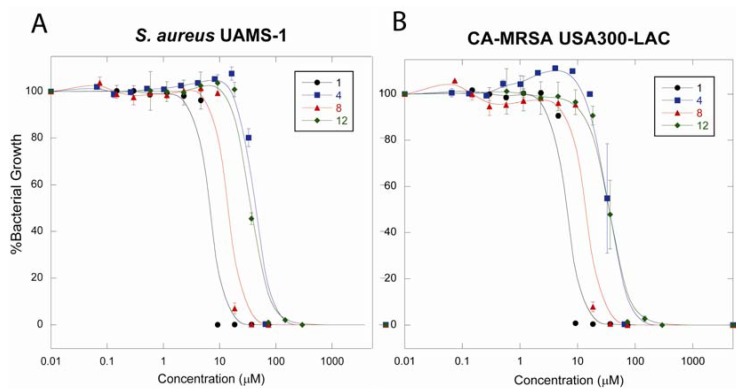
Dose-response curves show the antibacterial activities and reveal a structure-activity relationship for the chrysophaentins. Chrysophaentins A (**1**), D (**4**), and F (**8**), and fragment **12** were tested for their ability to inhibit the growth of *Staphylococcus aureus* UAMS-1 (panel **A**) and CA-MRSA USA300-LAC (panel **B**) in a microbroth dilution assay. Plots represent percentage bacterial growth as a function of compound concentration.

Growth curves for chrysophaentins A (**1**), D (**4**), and F (**8**), and compound **12** were graphed as percentage growth of untreated bacteria for *S. aureus* UAMS-1 (Figure 5A) and CA-MRSA USA300-LAC (Figure 5B). These curves clearly demonstrate the relationship between the structure of the chrysophaentins and analogs, halogen composition, and anti-staphylococcal activity.

### 2.5. Discussion

*C. taylori* is a rare marine chrysophyte alga. It was originally discovered in the Dry Tortugas off the southern coast of Florida [40], and has since been found in several locations worldwide. Bioactive natural products have been discovered from collections made in two locations. The Puerto Rico location yielded hormothamnione [41] and 6-desmethoxyhormothamnione [42], while the collections from St. John, U.S. Virgin Islands have yielded the ten chrysophaentins, as well as hormothamnione and 6-methoxyhormothamnione. As we have described here, analysis of methanol extracts revealed geographic variability within the *C. taylori* collections from St. John. The original collection coming from Round Bay in 2007 yielded chrysophaentins A–H. A second collection at Round Bay two years later contained a similar profile of compounds, indicating temporal stability in the natural compound production at this location. A 2009 collection at Long Bay contained the same eight chrysophaentins in a similar ratio. Both Long Bay and Round Bay are on the southeastern side of the island, and each of these collections was made in shallow protected waters, where the algae were growing on coral sand. The 2009 collection from Hawk’s Nest on the northwestern side of the island yielded a different profile of compounds. It was immediately obvious when comparing the chromatograms that the difference laid in the complete absence of the asymmetrical chrysophaentins in the samples collected at this location. Instead, the most abundant compounds included the acyclic chrysophaentin E, as well as two newly identified linear chrysophaentins, namely chrysophaentins E2 and E3.

Synthetic efforts to obtain a steady supply of chrysophaentin A are under way. An early approach that was employed provided two chrysophaentin fragments that we have shown exhibit similar potencies and spectrum of activity to those of the parent chrysophaentins. Previously, we profiled the anti-staphylococcal activity of several of the natural chrysophaentins towards common laboratory strains of *S. aureus*. In this study, we sought to expand the structure-activity relationship (SAR) by including the synthetic fragments, and to expand the number and diversity of *S. aureus* strains. We tested select compounds against five *S. aureus* strains to measure their potency (MIC_50_ and MIC_90_) and to determine whether the compounds were bacteriostatic or bactericidal. All chrysophaentins, natural and synthetic, were bacteriostatic at their MIC_90_; but were bactericidal at concentrations ranging from two to eight fold higher than their respective MIC_90_s. Furthermore, the synthetic fragments showed similar potency to the acyclic chrysophaentin E (**5**).

The synthetic fragments fit perfectly into our proposed structure-activity relationship. The tetrachlorinated macrocycle with the asymmetrical linkage between C-16 and C-1′ (chrysophaentin A) was essential for maximum potency. Compounds with an ether bond between C-14 and C-1′, which lead to a symmetrical macrocycle as seen in chrysophaentin F, show a decrease in overall potency. The presence of a fifth halogen at C-12′ did not decrease the antibacterial activity, even when the additional halogen was a bromine, indicating that the atom type at this position or even this ring is not critical to the observed activity. The absence of a macrocyclic structure, as in chrysophaentin E and the synthetic fragments **11** and **12**, further decreased antibacterial activity. However, the presence of the asymmetrical macrocycle did not guarantee potency as halogen diversity on the phenyl rings also affected activity. Chrysophaentin D, which contains two bromines in place of the chlorines on the phenol rings in **1**, was the least active compound considered in this study.

We have shown that subtle changes to the chrysophaentin structure and composition lead to differences in antibacterial activity. Knowing that the macrocycle is essential for full antibacterial potency, it is not surprising that the synthetic fragments described here have diminished activity compared to the cyclic natural products. However, we were gratified to find that they are indeed antimicrobial and their activities are comparable to the linear chrysophaentins. Their ease of synthesis and antimicrobial activity provide an excellent starting point not only toward the cyclic chrysophaentins, but also for generating more elaborate synthetic analogs.

The biosynthetic pathway leading to production of the chrysophaentins has not been characterized and a genome sequence for any *Chrysophaeum* species has yet to be published. Interestingly, the most closely related natural products include the marchantins, bazzanins, and chlorinated isoplagiochins, all of which were isolated from different species of liverworts [43]. While these macrocyclic compounds are reminiscent of the chrysophaentins, each family possesses defining structural characteristics that differ relative to one another and to the chrysophaentins. The marchantins [44] are bisbibenzyl ethers whose phenol rings are connected by an ethyl group, and thus far do not contain halogens. The bazzanins [45,46] and chlorinated isoplagiochins [47], on the other hand are chlorinated, but are bisbibenzyls rather than bisbibenzyl ethers. Like the marchantins and all liverwort metabolites, they also contain ethyl linkages. The presence of a butene linkage is unique to the chrysophaentins. While some information is known about the biosynthesis of the liverwort metabolites [48], the pathways are incompletely characterized; moreover, parallels to chrysophaentin biosynthesis cannot be predicted as the producing organisms are not closely related. The lack of biosynthetic information underlines the importance of a synthetic approach that could supply large quantities of the chrysophaentins for further biological studies.

## 3. Experimental Section

### 3.1. Biological Material

Samples of the marine algae were collected from Round Bay in July 2007, and Long Bay, Round Bay, and Hawk’s Nest in June 2009, St. John, U.S. Virgin Islands (Collection permit VIIS-2008-SCI-0034 National Park Service). Each of the alga samples were observed as fluffy, sulfur yellow colonies growing on coarse sand at shallow depths averaging −20 ft. The samples were identified as *Chrysophaeum taylori* Lewis and Bryan as described previously [27]. Briefly, the presence of clear stalk like structures made up of branching mucilaginous “streamers” of pear shaped, invaginated cells were visible by light microscopy (4–10×) on freshly collected specimens, as well as those preserved in 2.5% glutaraldehyde in sterile sea water immediately after collection.

### 3.2. LC-MS

Lyophilized material was sequentially extracted with hexanes, CH_2_Cl_2_, and MeOH. The MeOH extract was analyzed by reverse-phase HPLC with an MS detector (Jupiter Proteo C12, 250 × 4.6 mm, 4 µm, DAD at 220 and 280 nm, flow rate 0.5 mL/min) eluting with a linear gradient of 50–80% methanol in 0.05% TFA-water in 50 min.

### 3.3. Mass Spectrometry and MS/MS Fragmentation

The MeOH extract was partitioned between *n*-BuOH–H_2_O (1:1), and the organic layer was fractionated on Sephadex LH-20. Fractions containing the unknown chrysophaentins were subjected to HR-ESI-MS and MS in-source fragmentation. High resolution accurate mass data was obtained using a Waters LCT Premiere ESI-TOF mass spectrometer equipped with a Z-Spray electrospray ion source. The instrument was operated in the w-mode at a nominal resolution of 10,000 and an internal standard was used as reference. The ionization mode was negative ion and the ESI capillary voltage was −3000 V and the cone voltage was 30 V. The collisionally activated dissociation (CAD) mass spectra were obtained using a Waters LCT Premiere ESI-TOF mass spectrometer according to the method described by Ren *et al*. [49] The ion guide 1 voltage was 65 V and the cone voltage was 30 V. The instrument was operated in negative ion mode.

### 3.4. General Synthesis

All moisture sensitive reactions were performed using syringe-septum techniques under an atmosphere of either dry N_2_ or dry argon unless otherwise noted. All glassware was dried in an oven at 140 °C for a minimum of 6 h or flame-dried under an atmosphere of dry nitrogen prior to use. Reactions carried out at −78 °C employed a CO_2(s)_/acetone bath. Et_2_O and tetrahydrofuran were dried by distillation over sodium/benzophenone under an argon atmosphere. Dry methylene chloride was purified by filtration through an activated alumina column. All degassed solvents were prepared using the freeze/pump/thaw method (3×). Methanol, acetonitrile, and *N*,*N*-dimethylformamide were stored over molecular sieves (3 Å). Deuterated chloroform was stored over anhydrous potassium carbonate. Reactions were monitored by TLC analysis (pre-coated silica gel 60 F_254_ plates, 250 µm layer thickness) and visualized by using UV lamp (254 nm) or by staining with either Vaughn’s reagent (4.8 g of (NH_4_)_6_Mo_7_O_24_·4H_2_O and 0.2 g of Ce(SO_4_)_2_ in 100 mL of a 3.5 N H_2_SO_4_) or a potassium permanganate solution (1.5 g of KMnO_4_ and 1.5 g of K_2_CO_3_ in 100 mL of a 0.1% NaOH solution). Flash column chromatography was performed with 40–63 µm silica gel (Silicycle). Microwave reactions were performed on a Biotage Initiator microwave reactor. Infrared spectra were measured on a Smiths Detection IdentifyIR FT-IR spectrometer (ATR). Unless otherwise indicated, all NMR data were collected at room temperature in CDCl_3_ or (CD_3_)_2_CO on a 300, 400, 500, 600, or 700 MHz Bruker instrument. Chemical shifts (δ) are reported in parts per million (ppm) with internal CHCl_3_ (δ 7.26 ppm for ^1^H and 77.00 ppm for ^13^C), or internal acetone (δ 2.05 ppm for ^1^H and 29.85 ppm for ^13^C), as the reference. ^1^H NMR data are reported as follows: chemical shift, multiplicity (s = singlet, bs = broad singlet, d = doublet, t = triplet, q = quartet, m = multiplet, dd = doublet of doublets, dt = doublet of triplets, td = triplet of doublets, qd = quartet of doublets, sep = septet), integration, and coupling constant(s) (*J*) in Hertz (Hz).

3,5-Dimethoxy benzyl alcohol (**18**) [29]. To a stirred suspension of lithium aluminum hydride (13.1 g, 329 mmol, 2 equiv) in dry THF (400 mL) at 0 °C was added a solution of 3,5-dimethoxybenzoic acid (30.0 g, 164 mmol, 1 equiv) in dry THF (400 mL) over 45 min. Upon completion of the addition, the reaction mixture was diluted with THF (300 mL), warmed to rt, stirred for 5 h, and slowly quenched with a saturated aqueous solution of Na/K tartrate. The resulting biphasic mixture was stirred at r.t. for 1 h, the organic layer was separated, the remaining aqueous layer was extracted with EtOAc (×2), and the combined organic layers were dried (MgSO_4_), filtered, and concentrated under reduced pressure to give 27.0 g (97%) of **18** as a colorless oil: Rf 0.33 (EtOAc/hexanes, 1:2); IR (CDCl_3_) 3390, 2937, 1594, 1456, 1428, 1318, 1294, 1202, 1146, 1057, 829 cm^−1^; ^1^H NMR (600 MHz, CDCl_3_) δ_H_ 6.45 (d, 2 H, *J* = 2.4 Hz), 6.32 (d, 1 H, *J* = 2.4 Hz), 4.50 (d, 2 H, *J* = 5.4 Hz), 3.71 (s, 6 H), 3.49 (t, 1 H, *J* = 5.4 Hz); ^13^C NMR (150 MHz, CDCl_3_) δ_C_ 160.5, 143.3, 104.2, 99.2, 64.5, 55.0.

3,5-Dimethoxybenzyl bromide (**19**) [29]. To a stirred solution of **18** (17.5 g, 104 mmol, 1 equiv) in CH_2_Cl_2_ (500 mL) at 0 °C was added dropwise PBr_3_ (12.1 mL, 125 mmol, 1.2 equiv). The reaction mixture was slowly warmed to rt, stirred for 3 h, quenched with a saturated aqueous solution of NaHCO_3_, and stirred at r.t. for 1 h. The organic layer was separated and the aqueous layer was extracted with Et_2_O. The combined organic layers were dried (MgSO_4_), filtered, and concentrated under reduced pressure to give 22.2 g (92%) of **19** as a white solid: Rf 0.75 (EtOAc/hexanes, 1:2); IR (CDCl_3_) 2999, 2954, 1596, 1458, 1428, 1345, 1323, 1297, 1204, 1152, 1062, 930 cm^−1^; ^1^H NMR (400 MHz, CDCl_3_) δ_H_ 6.54 (d, 2 H, *J* = 2.0 Hz), 6.39 (d, 1 H, *J* = 2.4 Hz), 4.42 (s, 2 H), 3.80 (s, 6 H); ^13^C NMR (150 MHz, CDCl_3_) δ_C_ 160.9, 139.7, 107.0, 100.6, 55.4, 33.6; HRMS (ESI^+^) *m/z* calcd for C_9_H_12_O_2_Br 231.0021, found 231.0038.

(3-(3,5-Dimethoxyphenyl)prop-1-ynyl)trimethylsilane (**20**). To a stirred solution of ethynyl trimethysilane (39.1 mL, 277 mmol, 4 equiv) in THF (120 mL) at 0 °C was added ethylmagnesium bromide (3.16 M in Et_2_O; 87.6 mL, 277 mmol, 4 equiv). The reaction was warmed to rt, stirred for 30 min, and CuBr (9.93 g, 69.2 mmol, 1 equiv) was added. The reaction was stirred at r.t. for 15 min, 3,5-dimethoxybenzyl bromide (**19**, 16.0 g, 69.2 mmol, 1 equiv) was added, the resulting mixture was heated to 66 °C, and stirred overnight. The reaction mixture was diluted with Et_2_O, slowly quenched with brine, and extracted with Et_2_O (×2). The combined organic layers were washed with a saturated aqueous solution of NH_4_Cl, brine, dried (MgSO_4_), filtered, and concentrated under reduced pressure. The crude mixture was purified by chromatography on SiO_2_ (EtOAc/hexanes, 1:10) to give 17.2 g (100%) of **20 **as a pale yellow oil: Rf 0.41 (EtOAc/hexanes, 1:20); IR (neat) 2956, 2898, 2175, 1754, 1596, 1459, 1428, 1204, 1156, 1122, 1101, 839 cm^-1^; ^1^H NMR (400 MHz, CDCl_3_) δ_H_ 6.53 (d, 2 H, *J* = 2.4 Hz), 6.34 (t, 1 H, *J* = 2.4 Hz), 3.79 (s, 6 H), 3.60 (s, 2 H), 0.19 (s, 9 H); ^13^C NMR (100 MHz, CDCl_3_) δ_C_ 160.8, 138.7, 105.9, 104.0, 98.7, 87.1, 55.3, 26.3, 0.1; HRMS (ESI^+^) *m/z* calcd for C_14_H_21_O_2_Si 249.1311, found 249.1287.

1,3-Dimethoxy-5-(prop-2-ynyl)benzene (**15**). To a stirred solution of **20** (5.37 g, 21.6 mmol, 1 equiv) and AcOH (4.99 mL, 86.5 mmol, 4 equiv) in THF (100 mL) was added dropwise TBAF (1 M in THF; 86.5 mL, 86.5 mmol, 4 equiv). The resulting reaction mixture was stirred at r.t. for 24 h, diluted with Et_2_O, washed with brine (×2), dried (MgSO_4_), filtered, and concentrated under reduced pressure. The crude mixture was purified by chromatography on SiO_2_ (EtOAc/hexanes, 1:10) to give 3.85 g (100%) of **15** as a colorless oil: Rf 0.36 (EtOAc/hexanes, 1:10); IR (CDCl_3_) 3286, 2999, 2954, 1593, 1457, 1428, 1344, 1323, 1288, 1204, 1154, 1064, 827; ^1^H NMR (400 MHz, CDCl_3_) δ_H_ 6.53 (d, 2 H, *J* = 2.4 Hz), 6.35 (t, 1 H, *J* = 2.4 Hz), 3.79 (s, 6 H), 3.56 (d, 2 H, *J* = 2.8 Hz), 2.20 (t, 1 H, *J* = 2.8 Hz); ^13^C NMR (100 MHz, CDCl_3_) δ_C_ 160.9, 138.3, 105.9, 98.7, 81.7, 70.6, 55.3, 25.0; HRMS (EI^+^) *m/z* calcd for C_11_H_12_O_2_ 176.0837, found 176.0834.

5-(Prop-2-ynyl)-1,3-phenylene bis(2,2-dimethylpropanoate (**21**). To a stirred solution of **15** (6.50 g, 36.9 mmol, 1 equiv) in CH_2_Cl_2_ (1200 mL) at 0 °C was added BBr_3_ (1 M in CH_2_Cl_2_; 92.2 mL, 92.2 mmol, 5 equiv) over 1 h via an addition funnel. The reaction mixture was warmed to r.t. and stirred overnight. The reaction mixture was slowly quenched with a saturated aqueous solution of NaHCO_3_ (500 mL) and stirred at r.t. for 4 h. The solution was acidified with HCl, extracted with CH_2_Cl_2_ and EtOAc, and the combined organic layers were dried (MgSO_4_), filtered, and concentrated under reduced pressure. The crude mixture was dissolved in CH_2_Cl_2_ (180 mL), and triethylamine (20.9 mL, 148 mmol, 4 equiv) and PivCl (11.6 mL, 92.2 mmol, 2.5 equiv) were added. The resulting solution was stirred at r.t. for 90 min, diluted with brine, the organic layer was separated, and the aqueous layer was extracted with EtOAc. The combined organic layers were dried (MgSO_4_), filtered, and concentrated under reduced pressure. The crude mixture was purified by chromatography on SiO_2_ (EtOAc/hexanes, 1:10) to give 9.6 g (82%) of **21** as a colorless oil: Rf 0.85 (EtOAc/hexanes, 3:7); IR (neat) 3293, 2973, 1806, 1750, 1414, 1452, 1396, 1366, 1269, 1118, 1098, 1031, 1003 cm^−1^; ^1^H NMR (300 MHz, CDCl_3_) δ_H_ 6.95 (d, 2 H, *J* = 1.8 Hz), 6.76 (t, 1 H, *J* = 1.8 Hz), 3.61 (d, 2 H, *J* = 2.4 Hz), 2.21 (t, 1 H, *J* = 2.4 Hz); ^13^C NMR (75 MHz, CDCl_3_) δ_C_ 176.6, 151.5, 138.3, 118.3, 113.8, 80.7, 71.2, 39.1, 27.1, 24.5; HRMS (EI^+^) *m/z* calcd for C_19_H_24_O_4_ 316.1675, found 316.1670.

4-Chloro-2,5-dimethoxybenzaldehyde (**16**) [33,34]. To a stirred solution of 2-chloro-1,4-dimethoxybenzene (25.0 g, 0.145 mol, 1 equiv) and hexamethylene tetramine (20.5 g, 0.145 mol, 1 equiv) at r.t. was carefully added TFA (250 mL). The resulting yellow suspension was heated to 95 °C, stirred for 5 h, and the hot brown solution was poured into a 2 L Erlenmeyer flask containing approximately 250 g of crushed ice. To the vigorously stirred mixture was slowly added solid NaHCO_3 _(243 g, 2.90 mol, 20 equiv) in 5–10 g portions over two hours. The resulting yellow precipitate was filtered through a pad of celite, washed with water, and dissolved in Et_2_O. The organic layer was washed with water and brine, dried (MgSO_4_), filtered, and concentrated under reduced pressure to yield 29.0 g (100%) of **16** as an off-white solid: Rf 0.70 (EtOAc/hexanes, 3:7); IR (neat) 2941, 2874, 1664, 1601, 1575, 1497, 1478, 1461, 1389, 1269, 1213, 1023, 977 cm^−1^; ^1^H NMR (400 MHz, CDCl_3_) δ_H_ 10.35 (s, 1 H), 7.33 (s, 1 H), 7.03 (s, 1 H), 3.87 (s, 6 H); ^13^C NMR (100 MHz, CDCl_3_) δ_C_ 188.4, 156.1, 149.4, 130.4, 123.4, 114.5, 109.9, 56.5, 56.3; HRMS (EI^+^) *m/z* calcd for C_9_H_9_O_3_Cl 200.0240, found 200.0238.

(4-Chloro-2,5-dimethoxyphenyl)methanol (**25**). To a stirred solution of **16 **(29.0 g, 145 mmol, 1 equiv) in absolute ethanol (550 mL) was added sodium borohydride (27.3 g, 723 mmol, 5 equiv). The reaction mixture was stirred at r.t. for 6 h, quenched via dropwise addition of acetone, diluted with EtOAc, washed with brine (×2), dried (MgSO_4_), filtered, and concentrated under reduced pressure. The crude residue was purified by chromatography on SiO_2_ (EtOAc/hexanes, 1:1) to give 28.0 g (96%) of **25** as a white solid: mp 89–90 °C; Rf 0.53 (EtOAc/hexanes, 1:1); IR (neat) 3258, 2958, 2915, 1495, 1461, 1392, 1204, 1061, 719 cm^−1^; ^1^H NMR (300 MHz, CDCl_3_) δ_H_ 6.90 (s, 1 H), 6.80 (s, 1 H), 4.55 (d, 2 H, *J* = 4.2 Hz), 3.77 (s, 3 H), 3.71 (s, 3 H), 3.10 (bs, 1 H); ^13^C NMR (75 MHz, CDCl_3_) δ_C_ 150.6, 148.7, 128.4, 120.8, 112.5, 112.4, 60.3, 56.5, 55.6; HRMS (ESI^+^) *m/z* calcd for C_9_H_12_O_3_Cl 203.0475, found 203.0465.

1-(Bromomethyl)-4-chloro-2,5-dimethoxybenzene (**26**) [50]. To a stirred solution of **25** (5.00 g, 24.7 mmol, 1 equiv) in CH_2_Cl_2_ (125 mL) at 0 °C was added dropwise HBr (47–49% aqueous solution; 4.13 mL, 74.0 mmol, 3 equiv). The resulting solution was slowly warmed to r.t. and stirred overnight. The following morning a second batch of HBr (47–49% solution; 4.13 mL, 74.0 mmol, 3 equiv) was added to the reaction mixture, which was stirred at r.t. for an additional 4 h, extracted with Et_2_O (×2), washed with water, a saturated aqueous solution of NaHCO_3_, brine, dried (MgSO_4_), filtered, and concentrated under reduced pressure to give 6.55 g (100%) of **26** as a white solid: Rf 0.31 (EtOAc/hexanes, 3:7); IR (neat) 2962, 2947, 2844, 1732, 1582, 1495, 1458, 1443, 1389, 1301, 1204, 1182, 1033, 882 cm^−1^; ^1^H NMR (400 MHz, CDCl_3_) δ_H_ 6.91–6.90 (m, 2 H), 4.50 (s, 2 H), 3.84 (s, 3 H), 3.82 (s, 3 H); ^13^C NMR (75 MHz, CDCl_3_) δ_C_ 151.3, 148.9, 125.1, 123.2, 114.6, 113.6, 56.6, 56.2, 28.2.

(*E*)-5-(2-Chloro-3-iodoallyl)-1,3-phenylene bis(2,2-dimethylpropanoate (**22**) and (*Z*)-5-(2-Chloro-3-iodoallyl)-1,3-phenylene bis(2,2-dimethylpropanoate (**23**). To a stirred solution of **21** (9.50 g, 30.0 mmol, 1 equiv) in dry CH_2_Cl_2_ (150 mL) at 0 °C was added ICl (1 M in CH_2_Cl_2_; 30.0 mL, 30.0 mmol, 1 equiv). The reaction mixture was warmed to rt, stirred protected from light (enclosed in aluminum foil) for 3 h, diluted with Et_2_O, washed with Na_2_SO_4_ (×2), brine, dried (MgSO_4_), filtered, and concentrated under reduced pressure. The crude mixture was purified by chromatography on SiO_2_ (EtOAc/hexanes, 1:10) to give an inseparable 2:1 mixture of **22** and **23** (13.7 g, 95%) as a colorless oil: Rf 0.54 (EtOAc/hexanes, 1:10); IR (neat) 3277, 2934, 2872, 1746, 1592, 1497, 1461, 1409, 1269, 1207, 1122, 1103, 1032, 975, 723 cm^−1^; HRMS (ESI^+^) *m/z* calcd for C_19_H_24_O_4_NaClI 501.0306, found 501.0308. Characteristic data for the major, desired isomer **22**: ^1^H NMR (300 MHz, CDCl_3_) δ_H_ 6.87 (d, 2 H, *J* = 1.5 Hz), 6.80 (t, 1 H, *J* = 2.1 Hz), 6.62 (s, 1 H), 3.90 (s, 2 H), 1.35 (s, 18 H); ^13^C NMR (100 MHz, CDCl_3_) δ_C_ 176.6, 151.6, 137.9, 135.6, 119.0, 114.2, 74.8, 44.2, 39.1, 27.1. Characteristic signals for **23**: ^1^H NMR (300 MHz, CDCl_3_) δ_H_ 7.06 (s, 1 H), 3.92 (s, 2 H).

(*E*)-5-(2-Chloro-4-(4-chloro-2,5-dimethoxyphenyl)but-2-enyl)benzene-1,3-diol (**11**). A flame-dried microwave vial was charged with a 2:1 mixture of **22 **and **23 **(0.520 g, 1.08 mmol, 1 equiv), Pd(OAc)_2_ (0.0122 g, 0.0543 mmol, 0.05 equiv), P(*o*-tol)_3_ (0.0341 g (0.109 mmol, 0.1 equiv), and freshly distilled, dry, degassed DMF (0.8 mL). The reaction mixture was stirred at r.t. for 10 min. In a separate dry flask, a catalytic amount of iodine (~20 mg) and zinc (0.362 g, 5.43 mmol, 5 equiv) were heated (bunsen burner) until a purple gas coated the interior of the flask. The flask was cooled to rt, charged with distilled, dry, degassed DMF (1 mL), and benzyl bromide** 26 **(1.15 g, 4.34 mmol, 4 equiv). The mixture was allowed to stir at r.t. under argon for 6 min. The activated organozinc reagent was filtered through an oven dried fritted funnel under an atmosphere of argon and cannulated into the stirred solution of **22 **and **23**. The resulting solution was heated in a microwave reactor (2 min, 120 °C), treated with a second batch of Pd(OAc)_2_ (0.0122 g, 0.0543 mmol, 0.05 equiv) and resubjected to the microwave conditions (2 min, 120 °C). The crude reaction mixture was directly purified by chromatography on SiO_2_ (EtOAc/hexanes, 1:20) to give a crude yellow oil which was immediately dissolved in MeOH/CH_2_Cl_2_ (4 mL, 2:1), and Cs_2_CO_3_ (1.67 g, 5.08 mmol, 5 equiv) was added. The reaction mixture was stirred at r.t. for 6 h, diluted with EtOAc, and acidified with conc. HCl. The organic layer was separated, and the acidified aqueous solution was extracted with EtOAc. The combined organic layers were washed with brine, dried (MgSO_4_), filtered, and concentrated under reduced pressure. The crude mixture was purified by chromatography on SiO_2_ (chloroform/acetone, 8:2) to give a 2:1 mixture of **11 **and the undesired regioisomer **27** (0.192 g, 51%) as a yellow oil. Characteristic signals for **27**: ^1^H NMR (600 MHz (CD_3_)_2_CO) δ_H_ 8.20 (bs, 2 H), 7.02 (d, 2 H, *J* = 3.6 Hz), 6.45 (t, 1 H, *J* = 7.8 Hz), 6.26 (s, 3 H), 3.84 (app s, 5 H), 3.81 (s, 3 H), 3.50 (d, 2 H, *J* = 7.8 Hz); ^13^C NMR (150 MHz, CDCl_3_) δ_C_ 159.5, 152.3, 150.0, 141.5, 140.7, 127.7, 120.9, 115.6, 113.8, 108.1, 101.9, 101.7, 56.9, 56.5, 45.1.

An analytically pure sample of **11 **for biological evaluation and characterization was obtained via SFC chromatography using a semiprep (250 × 10 mm) silica column (Rt 5.80 min, 10 mL/min, 15% methanol, 220 nm detection): Rf 0.48 (acetone/chloroform, 2:8); IR (acetone) 3375, 3001, 2952, 1696, 1599, 1495, 1463, 1387, 1212, 1156, 1034, 1010 cm^−1^; ^1^H NMR (600 MHz (CD_3_)_2_CO) δ_H_ 8.18 (bs, 2 H), 7.02 (s, 1 H), 6.99 (s, 1 H), 6.30 (s, 2 H), 6.25 (s, 1 H), 5.88 (t, 1 H, *J* = 7.8 Hz), 3.84 (s, 3 H), 3.81 (s, 3 H), 3.73 (s, 2 H), 3.51 (d, 2 H, *J* = 7.8 Hz); ^13^C NMR (150 MHz, CDCl_3_) δ_C_ 159.5, 152.3, 150.0, 140.6, 133.7, 128.1, 128.1, 120.8, 115.5, 113.8, 108.0, 101.8, 56.9, 56.5, 40.2, 29.5; HRMS (ES^−^) [M + Cl]^−^ *m/z* calcd for C_18_H_18_O_4_Cl_3_ 403.0271, found 403.0295.

(*E*)-5-(2-Chloro-4-(4-chloro-2,5-dihydroxyphenyl)but-2-enyl)benzene-1,3-diol (**12**). A stirred solution of **11 **(0.0500 g, 0.135 mmol, 1 equiv) in CH_2_Cl_2_ (7 mL) was enclosed in aluminum foil and cooled to 0 °C. To the stirred solution was added dropwise BBr_3_ (1 M in CH_2_Cl_2_; 0.677 mL, 0.677 mmol, 5 equiv). The resulting solution was slowly warmed to r.t. and stirred overnight. The reaction mixture was quenched with a saturated aqueous solution of NaHCO_3_ (8 mL) and stirred at r.t. for an additional hour. The solution was acidified with HCl, extracted with EtOAc (×2) and the combined organic layers were dried (MgSO_4_), filtered, and concentrated under reduced pressure. The crude product was purified by chromatography on SiO_2_ (chloroform/acetone, 3:1) to give **12 **(0.0462 g, 96%) as a slightly yellow film. Before submission for biological testing, a sample of **12** was further purified via SFC chromatography using a semiprep (250 × 10 mm) silica column (Rt 4.68 min, 8 mL/min, 25% methanol, 220 nm detection): Rf 0.15 (acetone/chloroform, 2:8); IR (neat) 3343, 1692, 1599, 1495, 1417, 1329, 1184, 1143, 1005, 822 cm^−1^; ^1^H NMR (600 MHz (CD_3_)_2_CO) δ_H_ 8.17 (bs, 4 H), 6.86 (s, 1 H), 6.84 (s, 1 H), 6.29 (d, 2 H, *J* = 2.4 Hz), 6.24 (t, 1 H, *J* = 2.4 Hz), 5.89 (t, 1 H, *J* = 7.8 Hz), 3.69 (s, 2 H), 3.47 (d, 2 H, *J* = 7.8 Hz); ^13^C NMR (150 MHz, CDCl_3_) δ_C_ 159.5, 149.0, 146.9, 140.5, 133.8, 128.0, 127.0, 118.5 (2C), 116.7, 108.0, 101.8, 40.2, 29.5; HRMS (ES^−^) [2M − H]^−^ *m/z* calcd for C_32_H_27_O_8_Cl_4_ 679.0460, found 679.0482.

The demethylation reaction was also performed on a 2:1 mixture of **11 **and **27**, resulting in a 2:1 mixture of **12 **and its (*Z*)-diastereomer. Characteristic signals for the (*Z*)-diastereomer: ^1^H NMR (400 MHz (CD_3_)_2_CO) δ_H_ 6.85 (s, 1 H), 6.48 (t, 1 H, *J* = 7.6 Hz), 3.81 (s, 2 H), 3.46 (d, 2 H, *J* = 7.6 Hz).

### 3.5. Anti-Staphylococcal Activity

Microbroth dilution assays were performed as described in the CLSI guidelines. Minimum inhibitory concentrations (MIC_50_ and MIC_90_) were determined using 96-well microbroth dilution assays. Antimicrobial activity of pure compounds or antibiotic standards were tested by adding serial dilutions to wells containing Mueller Hinton II broth. An overnight growth of *S. aureus* ATCC 25923, MRSA ATCC BAA-41, MDRSA BAA-44, *S. aureus* UAMS-1, and CA-MRSA USA300-LAC was diluted in Mueller-Hinton II broth and added to the wells to a final concentration of 5 × 10^5^ colony forming units (CFU) per milliliter. Plates were incubated for 18 h at 37 °C with shaking at 200 rpm. Absorbance at 600 nm is read on a Molecular Devices plate reader. Growth curves are plotted, and curves were fit to a one-site model using the equation *y* = 100/[1 + (concentration/MIC_50_)], where MIC_50_ is the concentration at which the growth of bacterial cultures are reduced by 50%. MIC_90_ values were visually calculated by determining the lowest concentration of test compound that allowed no visible growth (no difference of absorption between treated samples and blank controls).

Bacteriostatic (BS) or bactericidal (BC) properties were determined by plating onto a fresh TSA plate an aliquot from each well that showed no visible growth starting at the MIC_90_. Plates were incubated for 24 h at 37 °C, and colonies were counted. Bactericidal concentrations were defined as those that resulted in a three logarithmic decrease in the number of viable bacteria relative to the starting inoculum, and the lowest bactericidal concentration was defined as the MBC.

## 4. Conclusions

The rare marine chrysophyte alga *C. taylori* has yielded a number of biologically active natural products. While the occurrence of *C. taylori* has been documented at a growing number of locations globally, thus far only collections obtained from the southeastern side of St. John appear to contain the full suite of chrysophaentins. The geographical variability described here, along with temporal and seasonal changes in the abundance of *C. taylori*, limited our ability to obtain additional material for study. Nevertheless, early synthetic efforts toward the synthesis of chrysophaentin A in particular have provided fragments of the natural products that have proven useful for biological studies. Here we have expanded the antimicrobial studies of several chrysophaentins and two synthetic fragments to include drug-susceptible and drug-resistant *S. aureus* strains that are of current clinical interest. Although approximately half the size of the cyclic chrysophaentins, we have shown that the potencies and spectrum of antimicrobial activity of the synthetic fragments are comparable to those of the parent chrysophaentins, in particular to the acyclic compound chrysophaentin E. Moreover, both the natural products and synthetic fragments are equally effective against both drug-resistant and drug-susceptible *S. aureus* strains. In summary, our results show promise for the notion of synthesizing equally potent chrysophaentin analogs that will be more readily available, and provide new data on the antimicrobial spectrum of this novel class of antibiotics.
